# Efficacy of Personalized Diabetes Self-care Using an Electronic Medical Record–Integrated Mobile App in Patients With Type 2 Diabetes: 6-Month Randomized Controlled Trial

**DOI:** 10.2196/37430

**Published:** 2022-07-28

**Authors:** Eun Young Lee, Seon-Ah Cha, Jae-Seung Yun, Sun-Young Lim, Jin-Hee Lee, Yu-Bae Ahn, Kun-Ho Yoon, Min Kyung Hyun, Seung-Hyun Ko

**Affiliations:** 1 Division of Endocrinology and Metabolism, Department of Internal Medicine, Seoul St. Mary's Hospital College of Medicine The Catholic University of Korea Seoul Republic of Korea; 2 Division of Endocrinology and Metabolism, Department of Internal Medicine, Wonkwang University Sanbon Hospital Gunpo Republic of Korea; 3 Division of Endocrinology and Metabolism, Department of Internal Medicine, St. Vincent’s Hospital College of Medicine The Catholic University of Korea Suwon Republic of Korea; 4 Catholic Institute of Smart Healthcare Center, The Catholic University of Korea Seoul Republic of Korea; 5 Department of Preventive Medicine, College of Korean Medicine Dongguk University Gyeongju Republic of Korea

**Keywords:** type 2 diabetes mellitus, digital health, mobile health, mHealth, mobile app, self-monitoring blood glucose, mobile phone

## Abstract

**Background:**

A system that combines technology and web-based coaching can help treat chronic conditions such as diabetes. However, the effectiveness of apps in mobile health (mHealth) interventions is inconclusive and unclear due to heterogeneous interventions and varying follow-up durations. In addition, randomized controlled trial data are limited, and long-term follow-up is lacking, especially for apps integrated into electronic medical records.

**Objective:**

We aimed to assess the effect of an electronic medical record–integrated mobile app for personalized diabetes self-care, focusing on the self-monitoring of blood glucose and lifestyle modifications, on glycemic control in patients with type 2 diabetes mellitus.

**Methods:**

In a 26-week, 3-arm, randomized, controlled, open-label, parallel group trial, patients with type 2 diabetes mellitus and a hemoglobin A_1c_ (HbA_1c_) level of ≥7.5% were recruited. The mHealth intervention consisted of self-monitoring of blood glucose with the automatic transfer of glucose, diet, and physical activity counseling data (iCareD system). Participants were randomly assigned to the following three groups: usual care (UC), mobile diabetes self-care (MC), and MC with personalized, bidirectional feedback from physicians (MPC). The primary outcome was the change in HbA_1c_ levels at 26 weeks. In addition, diabetes-related self-efficacy, self-care activities, and satisfaction with the iCareD system were assessed after the intervention.

**Results:**

A total of 269 participants were enrolled, and 234 patients (86.9%) remained in the study at 26 weeks. At 12 weeks after the intervention, the mean decline in HbA_1c_ levels was significantly different among the 3 groups (UC vs MC vs MPC: −0.49% vs −0.86% vs −1.04%; *P*=.02). The HbA_1c_ level decreased in all groups; however, it did not differ among groups after 26 weeks. In a subgroup analysis, HbA_1c_ levels showed a statistically significant decrease after the intervention in the MPC group compared with the change in the UC or MC group, especially in patients aged <65 years (*P*=.02), patients with a diabetes duration of ≥10 years (*P*=.02), patients with a BMI of ≥25.0 kg/m^2^ (*P*=.004), patients with a C-peptide level of ≥0.6 ng/mL (*P*=.008), and patients who did not undergo treatment with insulin (*P*=.004) at 12 weeks. A total of 87.2% (137/157) of the participants were satisfied with the iCareD system.

**Conclusions:**

The mHealth intervention for diabetes self-care showed short-term efficacy in glycemic control, and the effect decreased over time. The participants were comfortable with using the iCareD system and exhibited high adherence.

**Trial Registration:**

Clinical Research Information Service, Republic of Korea KCT0004128; https://tinyurl.com/bdd6pa9m

## Introduction

### Background

Diabetes is one of the most important chronic diseases that threatens public health [1]. Since 2000, the prevalence of diabetes has more than tripled, and by 2021, more than 530 million people worldwide will have diabetes [1]. The main goal of diabetes management is to maintain glycemic control within the target range, which is often accomplished through lifestyle modification and the self-monitoring of blood glucose (SMBG) in patients with type 2 diabetes mellitus (T2DM) [2,3]. However, maintaining glycemic control is challenging for both patients and health care providers (HCPs) because it is difficult to encourage or motivate patients to make long-term lifestyle changes, interpret their SMBG data, and provide immediate feedback and understand the patients’ lifestyle due to brief clinic visit times and long visit intervals. Digital health care or mobile health (mHealth) services facilitate the collection of personal data, analyze data to evaluate clinical conditions, and provide personalized interventions or monitoring [4]. Through mHealth systems, patients with T2DM are encouraged to consume a healthy diet and perform physical activity (PA). Patient-reported data are used to tailor feedback messages, including health promotion, motivation, encouragement, reminders, and emotional support messages. Therefore, mHealth interventions may improve the health outcomes in patients with T2DM via tailored personalized interventions [5].

Recent mHealth interventions targeting patients with T2DM have diverse goals and components, including insulin-management apps, wearable blood glucose meters, automated text messages, health diaries, and virtual health coaching [6]. However, the effect of apps on mHealth interventions remains inconclusive and unclear because of heterogeneous interventions and various lengths of follow-up. On the basis of a mixed treatment comparison network meta-analysis using data from published randomized controlled trials (RCTs), mobile apps or apps with e-coaching interventions for patients with T2DM were more effective in improving the hemoglobin A_1c_ (HbA_1c_) levels, fasting glucose, and hypoglycemia frequency than usual care (UC) during a 3- or 6-month follow-up period [7]. A meta-analysis of 13 studies on mobile apps for diabetes suggested overall efficacy in reducing HbA_1c_ levels, with a mean decrease of 0.44% (95% CI 0.29%-0.59%), as well as increased perception of self-care among mobile app users [8]. Various types of mHealth interventions have resulted in decrease in HbA_1c_ levels, in several RCTs that included patients with T2DM [9,10]. Most interventions demonstrated clinically and statistically significant efficacy, although some interventions had null results or achieved a <0.5% difference in the reduction of HbA_1c_ levels between the intervention and control groups [6].

Systems that combine technology and web-based coaching can be beneficial for treating diabetes and prediabetes [11,12]. To maintain lifestyle modification, patients should be continuously motivated and monitored in various ways, including individualized diabetes education and the use of aids based on information and communications technology [2,11]. Beyond using apps for personal use to collect and monitor lifelog data, web-based communication with physicians and HCPs would be more effective for diabetes self-care in patients with T2DM. Using this technology, physicians can view patient data in real time and between clinic visits and incorporate their lifelog data with clinical data derived from personal sensors and wearables in electronic medical records (EMRs). With regard to this type of intervention that connects a self-care app and EMRs, there is limited RCT data, and long-term follow-up is lacking.

### Objectives

Therefore, we designed a diabetes management system using an EMR-integrated mobile app that provided regular feedback from HCPs to support diabetes self-care in a clinical setting for patients with T2DM. The app mainly offers lifestyle counseling to aid the SMBG, diet planning, and PA. The purpose of this study was to compare the clinical efficacy of a 26-week personalized diabetes self-care system using an EMR-integrated mobile app with that of UC in patients with T2DM. We also compared the effectiveness of this system with and without feedback from the HCPs.

## Methods

### Study Design

**Figure 1:**
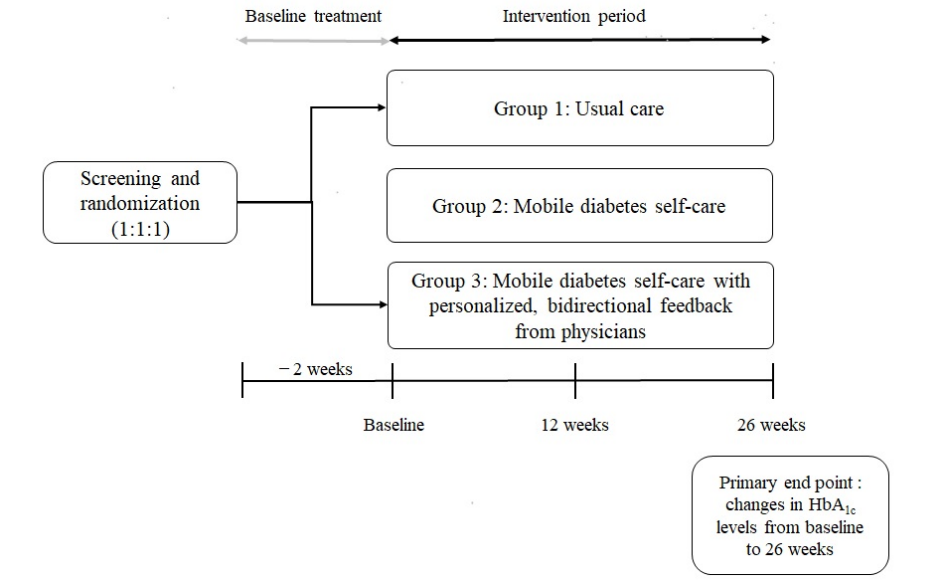
Study design. HbA_1c_: hemoglobin A_1c_.

**Figure 2:**
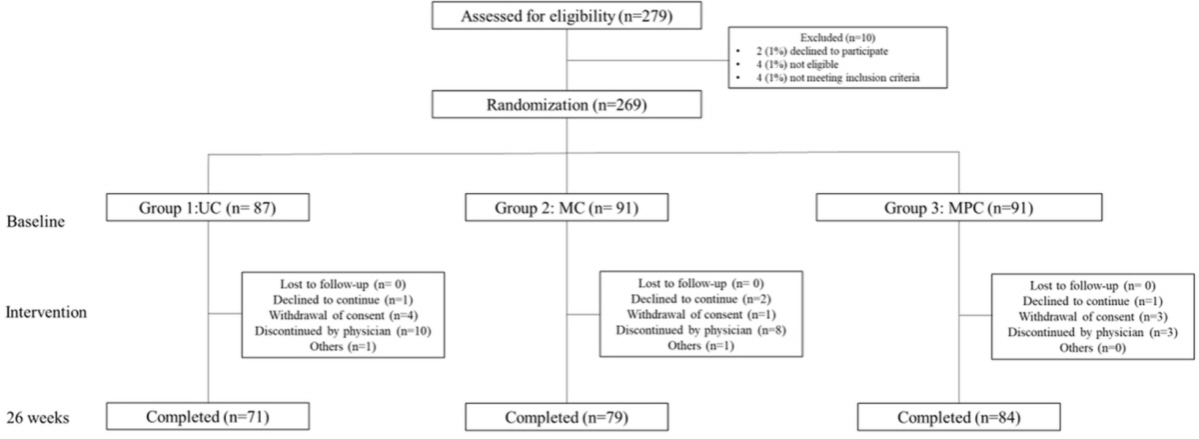
Flowchart of patient enrollment and status. MC: mobile diabetes self-care; MPC: mobile diabetes self-care with personalized, bidirectional feedback from physicians; UC: usual care.

This study was designed as a 26-week open-label, parallel group, 3-arm RCT conducted in 2 separate university-affiliated hospitals from August 2019 to December 2021. A detailed description of the study design has been previously reported [13]. As shown in Figure 1, all participants were randomly assigned to 1 of the following 3 groups: group 1, UC; group 2, mobile diabetes self-care (MC); and group 3, MC with personalized, bidirectional feedback from physicians (MPC). Among the 279 patients screened, 269 (96.4%) were enrolled in the study, and of these, 234 (86.9%) completed the intervention. A total of 269 individuals were included in the intention-to-treat (ITT) analyses (Figure 2).

### Eligibility

Patients aged 19 to approximately 74 years with T2DM, an HbA_1c_ level of ≥7.5%, and a BMI of ≥18.5 kg/m^2^ who could use a smartphone and consented to participate were eligible for this study. Participants who were using insulin pumps; who were pregnant; who had serious medical illness including end-stage renal disease, heart failure, and cancer; or who had difficulty performing PA were excluded. Further detailed inclusion and exclusion criteria are described in the study protocol [13]. The trial was registered with the Clinical Research Information Service, Republic of Korea (KCT0004128).

### Sample Size

On the basis of previous studies [10,14,15], we assumed a mean difference in HbA_1c_ levels of at least 0.60 between the control and intervention groups and an SD, within groups, of 0.75 after 6 months. In a single-factor ANOVA study, 73 patients per group resulted in 90% power and a .05 significance level. Finally, 282 participants (94 patients per group) were required to achieve 73 patients per group after accounting for a predicted dropout rate of up to 20%.

### Randomization

Those who signed the informed consent form were randomly assigned to 1 of 3 groups in a 1:1:1 ratio. Random numbers were generated using SAS (version 9.3; SAS Institute) [13]. Stratification by institution and a baseline HbA_1c_ level of 8.5% were performed using the stratified permutated block randomization method. As this study had an open-label design, HCPs and participants were informed of the group assignment at the time of randomization. The participants and HCPs could not be blinded in this study because of the intervention method.

### Intervention

Regardless of the assigned group, all participants were provided a glucometer (CareSens N; i-SENS, Inc) from which SMBG data were automatically transferred to our mHealth system, called the *iCareD system*. Basic diabetes education, including information on the SMBG, diet, and PA, was provided to all participants. The control group (UC) received UC according to the standard care for patients with T2DM by the Korean Diabetes Association [2]. Participants were instructed to perform the SMBG 4 times a day (before a meal in the morning and 2 hours after every meal), record their glucose levels in a notebook, and bring the notebook to their clinic visits. In the MC and MPC intervention groups, a diabetes self-care mobile app (iCareD; Medical Excellence Inc) was used in addition to the UC for diabetes management (Table S1 in Multimedia Appendix 1) [13]. The app allowed patients to enter their self-care data (SMBG, dietary habits, and step count), and automated text messages (educational, behavioral, and motivational messages) from the iCareD system were sent to their mobile phones. The automated messages were sent 3 times per week and consisted of 2 standardized messages for diabetes self-care and lifestyle modification and a customized message according to the lifestyle questionnaire. In the iCareD system, specific content for each message was developed based on the clinical practice guidelines of the Korean Diabetes Association and multidisciplinary expert opinions from our diabetes care team (endocrinologist, certified dietician, and diabetes educator). In both the MC and MPC groups, the mobile app was integrated with the EMR in each hospital; therefore, HCPs also evaluated participants at every 3-month visit based on the data obtained from the mobile app. Participants were instructed to upload their diet photos through the app. To encourage PA, we set the goal of a step count of >10,000 steps per day, and this goal was adjusted according to underlying diseases or individual health conditions. These data were also transferred to the iCareD system, which was integrated with the EMR system for HCPs in the hospital.

For the MPC group, based on our previous study, an HCP sent additional personalized recommendations and bidirectional feedback to each participant every 2 weeks through the iCareD system during the intervention period; the feedback was mainly related to diabetes self-care, the SMBG, or lifestyle modification [16].

In the offline system, patients visited the outpatient clinic every 3 months, and at these visits, physicians conducted face-to-face interviews with their patients, reviewed their uploaded data linked to the EMR, and provided individualized interventions based on these data. All participants were allowed to contact educator nurses over telephone but were encouraged to use the app.

### Primary and Secondary Outcomes

The primary outcome was the difference in the change in HbA_1c_ levels (%) between baseline and 26 weeks among the 3 groups. The secondary outcome was the changes in HbA_1c_ and fasting glucose (mg/dL) levels between the UC and 2 mobile-based intervention groups between baseline and 26 weeks. The HbA_1c_ level <7% attainment rates were evaluated at 12 and 26 weeks. In addition, lifestyle changes based on PA and diet records; cardiometabolic risk factors such as body weight, blood pressure, and lipid profile; program satisfaction and compliance (or adherence); frequency of hypoglycemia; and changes in homeostasis model assessment of insulin resistance and β cell function were assessed at 26 weeks. Adherence was defined as the proportion of intervention participation using the iCareD app, including blood glucose measurement and feedback confirmation, over a 24-week period. Exploratory assessment variables included changes in diabetes prescriptions, SMBG frequency, and BMI.

Participant satisfaction was assessed in the 2 intervention groups by using a locally developed satisfaction survey at 26 weeks. The survey included 5-level Likert-type questions evaluating self-care efficacy and various opinions on the iCareD system, such as the ease and frequency of text messages, perceived efficacy, and willingness to continue with or recommend the iCareD program to family or friends. A score of 5 indicated *very satisfied* or *strongly agree*. Higher scores on the satisfaction scale reflect better results.

### Measurements

Demographic and clinical information collected at baseline and follow-up has been described previously [13]. PA was tracked using a Google Fit mobile app and assessed as the total step count per day [17]. Body composition data were obtained using a bioimpedance analyzer (InBody 720 and 970, InBody Co, Ltd) at baseline and every 26 weeks. Laboratory parameters, including fasting glucose, HbA_1c_ level, and lipid profile, were collected at every visit. C-peptide and urinary albumin to creatinine ratios were measured at baseline and every 26 weeks. We used the updated homeostasis model assessment calculator to evaluate the homeostasis model assessment of insulin resistance and β cell function [18-20].

Hypoglycemic events, including hospitalization or emergency room visits due to hypoglycemia, blood glucose levels <70 mg/dL, or related symptoms even without the SMBG, were evaluated at every visit. Diabetes management behaviors such as SMBG frequency, PA, and diet records were obtained at every visit. SMBG frequency was defined as the average number of tests performed per day, calculated for each patient based on the records in the web system. The goal achievement rate for PA was defined as the number of days the target was reached/total measured days × 100 (%). User satisfaction with mobile app was surveyed in the MC and MPC groups.

### Statistical Analysis

Continuous variables were presented as mean (SD), whereas categorical data were presented as frequencies with percentages. Analysis of covariance was used to compare the mean 26-week HbA_1c_ levels among the 3 groups. Post hoc analysis was performed using the Bonferroni method. The number of hypoglycemic events among the groups was compared using the chi-square test or Fisher exact test. The goal achievement rate for PA was analyzed, except for the case of <1000 steps per day. Missing data were replaced by the last-observation-carried-forward method for all participants who were followed up at least once after enrollment. Both per-protocol and ITT analyses were conducted. Unless otherwise specified, analyses were performed based on the results of the ITT analysis. The analysis was performed using SAS (version 9.3; SAS Institute Inc). Statistical significance was set at *P* value of <.05.

### Ethics Approval

The study protocol was approved by the ethics committee of St. Vincent’s Hospital (VC19EEDI0085) and St. Mary’s Hospital (KC19EEDE0278). All participants provided written informed consent before enrollment in the study. All data and information were anonymized according to the International Conference on Harmonization Good Clinical Practice guidelines.

## Results

### Participant Flow

During the recruitment period from August 2019 to August 2020 in the outpatient clinics of 2 separate university-affiliated diabetes centers, a total of 279 participants were assessed for eligibility and 269 (96.4%) participants were randomized. A total of 10 participants withdrew consent, leaving 269 participants to be included in this study (Figure 2).

After the 26-week follow-up, the total retention rate was 86.9% (234/269), with an equal distribution among the groups. The baseline analysis revealed no significant differences between those who completed the study and those who were lost to follow-up (data not shown).

### Clinical Characteristics of Participants

The mean age of the participants was 52.5 (12.3) years, and 42.8% (115/269) of the participants were male. The mean baseline HbA_1c_ level and duration of diabetes were 8.7% (1.3%) and 11.4 (8.1) years, respectively. The mean BMI was 27.2 (4.6) kg/m^2^, and 41.3% (111/269) of the participants had hypertension. None of the other baseline characteristics or variables differed significantly among the 3 study groups. There was no significant difference in the presence of microvascular and macrovascular complications between the 3 groups (all *P*>.05; Table 1).

**Table 1:** Baseline demographic and clinical characteristics of patients.

		Group 1: UC^a^ (n=87)	Group 2: MC^b^ (n=91)	Group 3: MPC^c^ (n=91)	*P* value	
Age (years), mean (SD)		52.6 (12.1)	51.3 (13.1)	53.6 (11.7)	.66	
Sex (male), n (%)		37 (43)	40 (44)	38 (42)	.96	
Duration of diabetes (years), mean (SD)		11.5 (8.2)	10.9 (8.3)	11.9 (7.8)	.61	
Body weight (kg), mean (SD)		73.5 (17.2)	73.2 (16.9)	71.8 (13.0)	.97	
BMI (kg/m^2^), mean (SD)		27.4 (4.9)	27.3 (5.0)	26.8 (3.9)	.79	
SBP^d^ (mm Hg), mean (SD)		129.6 (14.5)	126.7 (15.0)	129.0 (14.5)	.38	
DBP^e^ (mm Hg), mean (SD)		78.0 (10.5)	77.1 (9.8)	76.8 (10.5)	.47	
Current smokers, n (%)		10 (12)	11 (12)	6 (7)	.40	
Alcohol consumption, n (%)		27 (31)	25 (28)	21 (23)	.49	
Physical activity (step count per day), mean (SD)		6280.9 (3159.4)	6208.9 (3212.8)	6630.0 (3639.8)	.78	
**Education, n (%)**						.25
	Elementary	10 (12)	5 (6)	14 (15)		
	High school	48 (55)	50 (55)	43 (47)		
	College	29 (33)	36 (40)	34 (37)		
**Comorbidities, n (%)**						
	Hypertension (yes)	39 (52)	36 (45)	36 (25)	.49	
	Hyperlipidemia (yes)	66 (88)	72 (90)	75 (89)	.92	
**Complication, n (%)**						
	CVD^f^	13 (15)	13 (14)	15 (17)	.91	
	Retinopathy	15 (17)	15 (17)	21 (23)	.46	
	Nephropathy	27 (31)	32 (35)	37 (41)	.40	
	Neuropathy	9 (10)	10 (11)	15 (17)	.40	
**Antidiabetic medications, n (%)**						.38
	Insulin only	1 (1)	3 (3)	2 (2)		
	Oral agents	53 (61)	50 (55)	46 (51)		
	Insulin+oral agents	23 (26)	28 (31)	35 (39)		
**Laboratory measurements, mean (SD)**						
	Fasting glucose (mg/dL)	164.7 (50.0)	163.6 (60.8)	166.3 (61.2)	.28	
	eGFR^g^ (ml/min/1.73 m^2^)	101.3 (23.3)	99.5 (22.9)	95.0 (28.4)	.08	
	HbA_1c_^h^ level (%)	8.6 (1.1)	8.7 (1.3)	8.8 (1.4)	.78	
	Total cholesterol (mg/dL)	154.7 (37.0)	170.2 (58.5)	157.7 (37.8)	.16	
	Triglyceride (mg/dL)	158.1 (107.0)	177.1 (254.0)	156.5 (111.4)	.72	
	HDL^i^ cholesterol (mg/dL)	48.2 (12.4)	50.3 (12.6)	48.2 (11.7)	.39	
	LDL^j^ cholesterol (mg/dL)	76.2 (32.4)	86.6 (35.6)	79.4 (29.9)	.13	
	C-peptide level (ng/mL)	2.1 (1.2)	2.2 (1.6)	2.1 (1.3)	.93	
	HOMA-IR^k^	1.9 (1.1)	1.9 (1.5)	1.9 (1.2)	.86	
	HOMA-β^l^	49.7 (32.7)	53.0 (36.2)	51.5 (38.0)	.74	

^a^UC: usual care.

^b^MC: mobile diabetes self-care.

^c^MPC: mobile diabetes self-care with personalized, bidirectional feedback from physicians.

^d^SBP: systolic blood pressure.

^e^DBP: diastolic blood pressure.

^f^CVD: cardiovascular disease.

^g^eGFR: estimated glomerular filtration rate.

^h^HbA_1c_: hemoglobin A_1c_.

^i^HDL: high-density lipoprotein.

^j^LDL: low-density lipoprotein.

^k^HOMA-IR: homeostasis model assessment for insulin resistance.

^l^HOMA-β: homeostasis model assessment for β-cell function.

### Primary Outcome Measure: Change in HbA_1c_ Level

**Figure 3:**
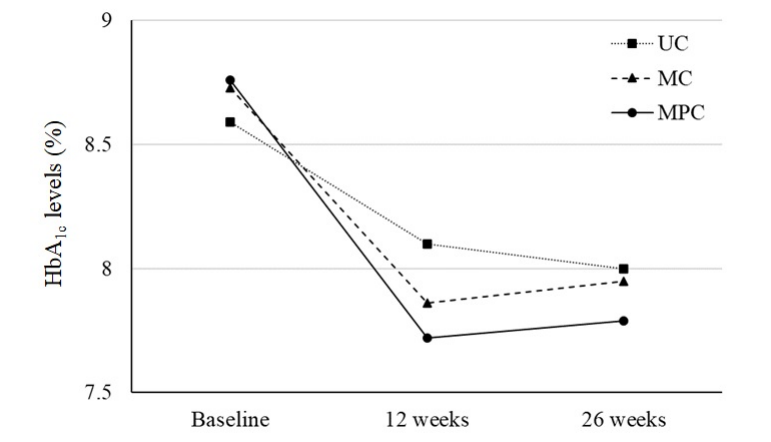
Mean HbA_1c_ level from baseline to 26 weeks. HbA_1c_: hemoglobin A_1c_; MC: mobile diabetes self-care; MPC: mobile diabetes self-care with personalized, bidirectional feedback from physicians; UC: usual care.

The change in HbA_1c_ levels did not differ significantly at 26 weeks among the 3 groups (Figure 3). However, the reduction in HbA_1c_ levels at 12 weeks was significantly different among the 3 groups (*P*=.02; Table 2). In the post hoc analysis, only the MPC group showed a significant decrease in HbA_1c_ levels compared with the UC group. (Table 2). In a subgroup analysis, a decrease in HbA_1c_ levels among the 3 groups showed a significant difference only at 12 weeks, especially in the patients aged <65 years (*P*=.02), patients with a diabetes duration of ≥10 years (*P*=.02), patients with a BMI of ≥25.0 kg/m^2^ (*P*=.004), patients with a C-peptide level of ≥0.6 ng/mL (*P*=.008), and patients who did not undergo treatment with insulin (*P*=.004; Table S2 in Multimedia Appendix 2). Adjusting for age, sex, and baseline HbA_1c_ level did not affect the HbA_1c_ level change results.

**Table 2:** Changes in hemoglobin A_1c_ (HbA_1c_) level from baseline and HbA_1c_ level <7% attainment rate.

			Group 1: UC^a^ (n=87)		Group 2: MC^b^ (n=91)		Group 3: MPC^c^ (n=91)		*P* value
**Changes in HbA_1c_ level from baseline (%), mean (SD)**									
	Change at 12 weeks	−0.5 (1.0)		−0.9 (1.4)		−1.0 (1.5)^d^		.02	
	Change at 26 weeks	−0.6 (1.1)		−0.8 (1.7)		−1.0 (1.5)		.30	
**HbA_1c_ level <7% attainment rate, n (%)**									
	At 12 weeks	12 (14)		15 (17)		21 (23)		.25	
	At 24 weeks	15 (17)		15 (17)		26 (29)		.08	

^a^UC: usual care.

^b^MC: mobile diabetes self-care.

^c^MPC: mobile diabetes self-care with personalized, bidirectional feedback from physicians.

^d^*P*<.05 versus group 1 in post hoc analysis.

### Secondary Outcome Measures

The HbA_1c_ level <7% attainment rate increased from between the UC and MPC groups at 12 weeks, but the difference was not significant (UC vs MC vs MPC: 12/87, 14% vs 15/91, 17% vs 21/91, 23%; *P*=.25). The MPC group tended to have higher attainment rate of HbA_1c_ level <7% at 26 weeks, compared with the other groups (UC vs MC vs MPC: 15/87, 17% vs 15/91, 17% vs 26/91, 29%; *P*=.08; Table 2). Other changes in clinical and behavioral outcomes from baseline to follow-up are shown in Table 3. Fasting glucose levels were significantly reduced from baseline to each time point in all 3 groups (*P*<.05 for each group, each follow-up time). However, the change in fasting glucose levels did not differ among the 3 groups during the 26-week intervention period (Table 3). Changes in body weight and BMI from baseline to 26 weeks also showed no differences among the study groups.

The frequency of the SMBG did not show any significant differences among the 3 groups at the 26-week follow-up. However, compared with patients in the UC group, those in the 2 intervention groups (iCareD system users) tended to have more frequent SMBG recordings at 12 weeks (UC vs iCareD system users: 1.4 [1.1] times per day vs 1.6 [1.0] times per day; *P*=.09). The average frequency of the SMBG showed a negative correlation with HbA_1c_ (*r*=0.277; *P*=.003).

PA, defined as step counts per day, was not significantly different among the study groups 26 weeks after the intervention. The goal achievement rate for PA was higher in the MC and MPC groups than that in the UC group at 26 weeks, but the difference was not significant (UC vs MC vs MPC: 15.1% vs 18.5% vs 17.9%; *P*=.45). The changes in low-density lipoprotein–cholesterol level showed significant differences among the 3 groups at both 12 and 26 weeks (*P*=.001 for 12 weeks and *P*=.02 for 26 weeks). Low-density lipoprotein–cholesterol levels increased in the UC group and decreased in the MC and MPC groups during the follow-up period.

**Table 3:** Secondary study outcomes at baseline and follow-up.

		Group 1: UC^a^ (n=87)	Group 2: MC^b^ (n=91)	Group 3: MPC^c^ (n=91)	*P* value
**Fasting glucose (mg/dL), mean (SD)**					
	Baseline	164.7 (50.0)	163.6 (60.8)	166.3 (61.2)	.83
	12 weeks	154.6 (54.7)	145.3 (51.7)	146.3 (45.3)	.28
	26 weeks	148.9 (55.7)	146.3 (56.2)	142.7 (47.5)	.76
	Change from baseline at 12 weeks	−10.1 (50.9)	−18.3 (64.3)	−20.0 (50.6)	.68
	Change from baseline at 26 weeks	−15.8 (57.0)	−17.3 (64.5)	−23.7 (57.6)	.89
**PA^d^ (step counts/day), mean (SD)**					
	Baseline to approximately 12 weeks	6069.3 (2774.4)	6143.0 (2849.6)	6447.0 (3338.2)	.87
	12 weeks to 26 weeks	5827.0 (2879.9)	6019.3 (2953.1)	6319.1 (3652.4)	.94
**Body weight (kg), mean (SD)**					
	Baseline	73.5 (17.2)	73.2 (16.9)	71.9 (13.0)	.97
	12 weeks	73.2 (17.3)	73.0 (17.1)	71.8 (13.2)	.98
	26 weeks	73.1 (17.1)	73.2 (17.4)	71.8 (13.2)	.97
	Change from baseline at 12 weeks	−0.27 (1.85)	−0.19 (2.05)	−0.07 (2.17)	.93
	Change from baseline at 26 weeks	−0.42 (2.84)	0.03 (3.22)	−0.03 (3.51)	.98
**BMI (kg/m^2^), mean (SD)**					
	Baseline	27.4 (4.8)	27.3 (5.0)	26.9 (3.9)	.80
	12 weeks	27.3 (4.8)	27.2 (5.1)	26.8 (4.0)	.84
	26 weeks	27.3 (4.7)	27.3 (5.2)	26.8 (4.0)	.91
	Change from baseline at 12 weeks	−0.11 (0.73)	−0.07 (0.72)	−0.03 (0.82)	.94
	Change from baseline at 26 weeks	−0.18 (1.08)	0.01 (1.19)	−0.01 (1.33)	.97
**LDL^e^ cholesterol (mg/dL), mean (SD)**					
	Baseline	76.2 (32.4)	86.6 (35.6)	79.4 (29.9)	.13
	12 weeks	80.5 (36.0)	80.3 (34.6)	76.2 (33.8)	.52
	26 weeks	78.6 (33.0)	77.5 (33.7)	76.8 (34.7)	.86
	Change from baseline at 12 weeks	4.8 (19.3)	−6.4 (29.2)	−5.1 (25.8)	.001
	Change from baseline at 26 weeks	3.5 (18.7)	−9.1 (32.0)	−4.8 (30.3)	.02
**HOMA-IR^f^, mean (SD)**					
	Baseline	1.9 (1.1)	1.9 (1.5)	1.9 (1.2)	.88
	26 weeks	1.8 (1.0)	1.9 (1.5)	1.8 (1.4)	.76
	Change from baseline at 26 weeks	−0.1 (0.8)	0.0 (0.1)	−0.2 (1.2)	.81
**HOMA-β^g^, mean (SD)**					
	Baseline	49.7 (32.7)	53.0 (36.2)	51.5 (38.0)	.74
	26 weeks	58.5 (30.2)	60.5 (35.3)	67.7 (80.6)	.99
	Change from baseline at 26 weeks	8.6 (28.2)	6.9 (37.5)	14.5 (75.5)	.78

^a^UC: usual care.

^b^MC: mobile diabetes self-care.

^c^MPC: mobile diabetes self-care with personalized, bidirectional feedback from physicians.

^d^PA: physical activity.

^e^LDL: low-density lipoprotein.

^f^HOMA-IR: homeostasis model assessment for insulin resistance.

^g^HOMA-β: homeostasis model assessment for β-cell function.

### Satisfaction for iCareD System

A total of 157 out of the 182 (86.2%) participants completed the satisfaction survey at 26 weeks of the intervention. Participants’ satisfaction with the iCareD program was very high in both the MC and MPC groups. Overall, most patients were satisfied with the system (104/157, 66.2% strongly agree; 33/157, 21% agree), understood all the messages (27/157, 17% strongly agree; 89/157, 57% agree), were willing to use the program (56/157, 36% strongly agree; 73/157, 46% agree), felt that it helped them reach their goals (74/157, 47% strongly agree; 61/157, 39% agree), and recommended the iCareD system to a family member or friend with T2DM (57/157, 36% strongly agree; 77/157, 49% agree). No differences were observed between the 2 groups (Table 4).

**Table 4:** Messages read for 6 months (intervention period) and program satisfaction.

			Group 2: MC^a^ (n=91)		Group 3: MPC^b^ (n=91)		*P* value
Automated message sent, N			141		155		N/A^c^
Automated message read, n (%)			110 (78)		134 (86.4)		.09
Personalized message sent, N			N/A		12		N/A
Personalized message read, n (%)			N/A		12 (100)		<.001
**Satisfaction survey**							
	Total, N	77		80		N/A	
	Overall satisfaction, n (%)	67 (87)		70 (88)		.39	
	Help to diabetes self-care, n (%)	67 (87)		69 (86)		.37	
	Will you recommend the app to others? n (%)	67 (87)		67 (84)		.92	
	Do you want to continue using the app? n (%)	63 (81)		66 (83)		.70	

^a^MC: mobile diabetes self-care.

^b^MPC: mobile diabetes self-care with personalized, bidirectional feedback from physicians.

^c^N/A: not applicable.

There were no statistically significant differences between the MC and MPC groups in skill and technique acquisition, health service navigation, or manipulation of app content. iCareD system users especially valued convenient SMBG data reporting via automatic wireless transfer from the glucometer to the app without needing to write directly in the notebook and the ability to browse accumulated personal data (63/157, 40% strongly agree; 69/157, 44% agree). An end-of-intervention usability survey demonstrated that participants were comfortable with using the iCareD system.

With regard to adherence, compared with participants in the MC group, those in the MPC group checked the automated text messages from the iCareD system for 26 weeks. The proportion of participants who read >75% of the automated messages (3 times per week) was significantly higher in the MPC group than in the MC group (76/91, 83% vs 61/91, 67%; *P*=.02). The number of participants who uploaded photos of a meal and step count was higher in the MPC group than in the MC group, but the proportion declined to 50% by the end of the study in both groups.

### Adverse Events

No serious adverse events were reported from enrollment until the completion of this study. Hypoglycemic events were infrequent and showed no differences among the groups at 26 weeks (Table 5). No deaths, direct study-related adverse events, or severe hypoglycemic episodes were reported or detected.

**Table 5:** Hypoglycemic events.

			Group 1: UC^a^		Group 2: MC^b^		Group 3: MPC^c^		*P* value
**Patients who experienced hypoglycemia, N**			87		91		91		N/A^d^
	Baseline to <12 weeks, n (%)	15 (17)		34 (35)		32 (35)		.03	
	12 weeks to 26 weeks, n (%)	22 (25)		28 (34)		26 (30)		.84	
**Frequency of hypoglycemia^e^, mean (SD)**									
	Baseline to <12 weeks	6.1 (7.4)		2.9 (3.1)		4.3 (5.6)		.25	
	12 weeks to 26 weeks	3.2 (4.1)		3.3 (2.9)		3.6 (3.8)		.56	
Unexpected clinic visit or hospitalization due to hypoglycemia^f^, n (%)			0 (0)		2 (2)		0 (0)		.18

^a^UC: usual care.

^b^MC: mobile diabetes self-care.

^c^MPC: mobile diabetes self-care with personalized, bidirectional feedback from physicians.

^d^N/A: not applicable.

^e^Per patient who experienced hypoglycemia for 90 days.

^f^Hypoglycemia was defined as a blood glucose level of <70 mg/dL.

## Discussion

### Principal Findings

This study was an RCT investigating a hospital-based, EMR-integrated mobile app–based diabetes self-care intervention over a 26-week period in patients with T2DM. Although the HbA_1c_ level decreased from the baseline value in all 3 groups, the HbA_1c_ changes did not show any significant difference between the control and 2 intervention groups at 26 weeks. However, we found that interactive mHealth intervention for diabetes self-care (MPC group) significantly decreased HbA_1c_ levels by −1.04% compared with −0.49% in the UC group and −0.86% in the MC group at 12 weeks. This finding was consistent among non–insulin users, those aged < 65 years, and those who were obese.

Owing to the global growth in the use of mobile phones with powerful platforms to help health care, many types of apps have been developed. According to Liquid-State, in 2018, there were >318,000 mHealth care apps available for patients, and approximately 200 new health care apps were being built each day [21]. In all, 70% of mHealth practitioners have reported that diabetes is currently the leading health target for the mobile app industry [22]. In 2017, more than 1500 diabetes-related apps were reported to be available to users.

Mobile apps related to diabetes management generally deal with information about diabetes, healthy diet, PA, weight loss, the SMBG, adherence, and motivation [6]. mHealth interventions support self-care and diabetes education and encourage lifestyle modification. These data may be used to tailor feedback messages or advice on specific behavior changes to implement; these messages are usually sent automatically according to an algorithm [5,9,23]. Compared with conventional mobile apps that collect only patient-driven data, our EMR-integrated mobile app could provide important clues to the future direction of mobile app development for diabetes management in 2 respects. First, HCPs can be provided with patients’ medical history such as comorbidities or current medications, in addition to patient-centered data. Given the high rates of comorbidity and concurrent medications in patients with T2DM [24], this integrated provision of medical information may allow HCPs to provide accurate guidance to patients on diet, exercise, and management of comorbid diseases rather than simply focusing on the message to lower blood glucose levels. Second, from the patients’ perspective, it is possible to provide better insight into diabetes management by providing laboratory results by time course along with personal data collection information. In particular, our systems adopted visualization of glucose levels by color to improve awareness or alertness of hyperglycemia (red) or hypoglycemia (black) [13].

Using the EMR-integrated mobile app intervention, we demonstrated a significant reduction in HbA_1c_ levels after 12 weeks of intervention. Consistent with our results, a 3-month RCT using DialBetics, a smartphone-based self-management support system for Japanese patients with T2DM, demonstrated that HbA_1c_ levels decreased by an average of 0.4% compared with an increase of 0.1% in the control group, with improvement in fasting glucose level and BMI [25]. A systematic review also revealed limited robust evidence of the promising short-term effectiveness of mHealth interventions for diabetes, such as the improvement of HbA_1c_ levels [14,15,26,27]. However, a caveat of these RCT analyses is that most of them included only studies conducted under highly controlled conditions with a small number of patients [14,15,26,27]. Interestingly, the first Food and Drug Administration–approved mobile app, BlueStar, showed no intervention effects in a real-world setting with >100 patients, despite significant reductions in HbA_1c_ (>1%) in their first RCT with 30 patients [28,29]. It is noteworthy that our study showed significant differences in HbA_1c_ levels among groups at 3 months in real-world practice, with a relatively large number of patients at 2 different clinical sites. This finding suggests the potential usefulness of EMR-integrated mobile app interventions in diabetes management. In addition, we found that the intervention effects in the MPC group were prominent in patients with younger age, obesity, higher C-peptide levels, and no insulin treatment. This finding implies that mobile-based interventions, such as other diabetes treatments, may be more effective when β cell function is preserved. This also highlights the importance of early intervention. However, the intervention effect was also pronounced in those with a diabetes duration ≥10 years. This indicates that although early intervention may be important, such interventions may also be effective in long-standing diabetes.

Mobile phone apps that receive blood glucose data from a connected glucometer are available and have the capacity to make data upload and review less burdensome [30]. The internet-based SMBG system, which augments the SMBG by giving patients the means to communicate their blood glucose levels to their HCP for actional feedback, has been shown to reduce HbA_1c_ levels in some RCTs involving patients with T2DM [31]. The inverse correlation between reporting frequency and HbA_1c_ levels, as well as the significant difference in HbA_1c_ levels only for frequent testers (defined as those who test on average twice or more per day), suggests that frequent SMBG has an effect on reducing HbA_1c_ levels only when combined with regular, frequent communication of SMBG with an HCP [32]. The recording of a food diary using a smartphone app is a well-known simple tool, and technology to use images to quantify the composition and calorie content of food has been developed. However, it is difficult and cumbersome for users to constantly record data based on their eating habits [33,34]. However, automated integration of glucose and lifelog data in the EMR between scheduled clinic visits improves the HCP workflow for reviewing data and improves communication with patients, eventually leading to better care [35].

In this study, 87.2% (137/152) of the participants were satisfied with the iCareD system and answered that the app helped their diabetes self-care skills; however, the iCareD system failed to decrease HbA_1c_ levels over >3 months. There are possible explanations for the lack of improvement in HbA_1c_ levels in mHealth app users. First, age is a barrier to digital health care adoption and may influence the adoption of new technologies [36]. The mean age of the participants in this study was 52.5 years (range 20-74 years). A total of 16% (42/269) of the participants were aged >65 years. Second, the iCareD system was developed with a focus on lifestyle changes rather than strict glucose control or active medication adjustment, such as whirlwind dosage escalation of antidiabetic medications. In the case of the TExT-MED study, a unidirectional text message intervention for diabetes self-care providing text message triggers to encourage individuals to engage in self-care behaviors, the TExT-MED program also did not result in a significant improvement in HbA_1c_ levels. However, trends toward improvement in the primary outcome of HbA_1c_ levels and other secondary outcomes, including quality of life, were observed. Similar to our satisfaction survey, 94% (44/47) of the patients who received the TExT-MED intervention enjoyed the program and believed it was a good way to learn about diabetes [5]. Patient engagement was highest for more medical topics, such as glucose monitoring and medications, and lower for lifestyle topics, such as PA and healthy coping [6]. Therefore, we suggest that interventions for diabetes self-care should include improving HbA_1c_ levels through modification of lifestyle, glucose monitoring, and adherence and dosage adjustment for antidiabetic medications [37]. Third, our patients had a long duration of diabetes and were insulin users [32]. In general, the effects of education and lifestyle changes decrease with the duration of diabetes [38]. Fourth, there was no evidence of the most effective frequency of the intervention messages. We sent personalized intervention messages from HCPs every 2 weeks and automated general informative messages every other day. Patient satisfaction and accessibility are important for improving self-management efficiency, and the clinical course can be improved through personalized intervention [4]. More frequent, bidirectional, real-time communication with HCPs and patients would lead to more effective improvement in HbA_1c_ levels.

Although we did not observe remarkable improvement in HbA_1c_ levels over the long term, it is encouraging that the goal achievement rates for PA were higher in the intervention group at 26 weeks. When the target of 7500 steps per day was applied [39-41], the difference in goal achievement rates among the groups further increased (UC vs MC vs MPC: 25.9% vs 28.5% vs 30.2%). Given the lifelong management of T2DM, the small differences observed in the short term may increase in the future. Furthermore, in terms of the prevention of diabetic complications such as cardiovascular disease, PA cannot be overemphasized [40-42]. Finally, we expect that our study will provide more solid evidence of the short-term efficacy of mobile app–based diabetes management. In particular, in relation to the recent global public health crisis, the COVID-19 pandemic, this methodology is expected to contribute greatly in the future to promote the rapid introduction and diffusion of new digital health–related technologies such as telemedicine [43].

To maximize the effect of mHealth interventions, it is important to tailor the intervention in a patient-centered manner and evaluate user satisfaction [44]. Undoubtedly, more RCTs with longer follow-up periods should be conducted to evaluate the long-term effects of diabetes-related mobile apps and to confirm that the outcomes seen in initial studies are sustainable over time [22].

### Conclusions

In summary, the use of iCareD apps for diabetes self-care can be considered an effective measure, especially when patients can communicate with HCPs [8]. Remote health data monitoring and real-time communication with patients supported self-care of diabetes, resulting in short-term improvement in HbA_1c_ levels. An mHealth system for patients with T2DM should be developed to support and motivate sustainable behavior changes in patients and to allow for an approach that is more tailored to individual needs.

## Appendix

Multimedia Appendix 1 Comparison of the intervention protocol in each group.

Multimedia Appendix 2 Hemoglobin A_1c_ (HbA_1c_) level and HbA1c level changes from baseline to 12 and 26 weeks according to subgroup analysis.

Multimedia Appendix 3 CONSORT-eHEALTH checklist (V 1.6.1).

## Abbreviations

EMR: electronic medical record
HbA_1c_: hemoglobin A_1c_
HCP: health care provider
ITT: intention-to-treat
MC: mobile diabetes self-care
mHealth: mobile health
MPC: mobile diabetes self-care with personalized, bidirectional feedback from physicians
PA: physical activity
RCT: randomized controlled trial
SMBG: self-monitoring of blood glucose
T2DM: type 2 diabetes mellitus
UC: usual care
