# Supplementary material for: Efficacy of Personalized Diabetes Self-care Using an Electronic Medical Record–Integrated Mobile App in Patients With Type 2 Diabetes: 6-Month Randomized Controlled Trial
Source: J Med Internet Res. 2022 Jul 28;24(7):e37430. doi: 10.2196/37430 (PMC9496112; doi:10.2196/37430)
Supplement: Multimedia Appendix 1 [file jmir_v24i7e37430_app1.docx]

**Table S1.** Comparison of the intervention protocol in each group

|  | Control | Intervention | |
| --- | --- | --- | --- |
|  | Group 1: UC^a^ | Group 2: MC^b^ | Group 3: MPC^c^ |
| Education | Comprehensive management of diabetes including self-care | Comprehensive management of diabetes including self-care | Comprehensive management of diabetes including self-care |
| Instruction | Do SMBG^d^ (4 times a day) | Do SMBG^d^ (4 times a day),  upload diet photos | Do SMBG^d^ (4 times a day),  upload diet photos |
| Monitoring | SMBG^d^ note, lifestyle* questionnaire, laboratory data | SMBG^d^ and lifestyle* log on web-based system, individualized monthly reports about comprehensive management, laboratory data | SMBG^d^ and lifestyle* log on web-based system, individualized monthly reports about comprehensive management, laboratory data |
| Intervention | Usual care only | Usual care with mobile diabetes self-care | Usual care with mobile diabetes self-care |
| Message categories | - | Motivational and encouraging: EOD^e^  SMBG^d^: weekly  Healthy eating: EOD^e^  Active behavior and physical activity: EOD^e^ | |
| Feedback from physicians | During visit (every 13 weeks) | During visit (every 13 weeks) | During visit (every 13 weeks) and between visits (every 2 weeks) through mobile app |
| Immediate intervention between visits | Impossible | Impossible | Possible |
| Patient-Physician bidirectional communication between visits | No | No | Yes |

^a^UC: usual care.

^b^MC: mobile diabetes self-care.

^c^MPC: mobile diabetes self-care with personalized, bidirectional feedback from physicians

^d^SMBG: self-monitoring blood glucose

^e^EOD: every other day
