# Supplementary material for: Efficacy of Personalized Diabetes Self-care Using an Electronic Medical Record–Integrated Mobile App in Patients With Type 2 Diabetes: 6-Month Randomized Controlled Trial
Source: J Med Internet Res. 2022 Jul 28;24(7):e37430. doi: 10.2196/37430 (PMC9496112; doi:10.2196/37430)
Supplement: Multimedia Appendix 2 [file jmir_v24i7e37430_app2.docx]

**Table S2.** A_1C_ level and A_1C_ level changes from baseline to 12 and 26 weeks according to subgroup analysis.

|  | Age <65 years | | | | Age ≥65 years | | | |
| --- | --- | --- | --- | --- | --- | --- | --- | --- |
|  | Group 1 (n=74) | Group 2 (n=78) | Group 3 (n=75) | *P* value | Group 1 (n=13) | Group 2 (n=13) | Group 3 (n=16) | *P* value |
|  |  |  |  |  |  |  |  |  |
| 12 weeks, mean (SD) | 8.1 (1.2) | 8.0 (1.2) | 7.8 (1.1) | .18 | 8.2 (1.3) | 7.3 (0.9) | 7.6 (0.7) | .09 |
| 26 weeks, mean (SD) | 8.0 (1.3) | 8.1 (1.6) | 7.9 (13) | .59 | 8.0 (1.3) | 7.2 (0.6) | 7.4 (0.5) | .06 |
| Change at 12 weeks, mean (SD) | −0.6 (0.9) | −0.9 (1.5) | −1.1 (1.5) | .02 | −0.1 (1.1) | −0.9 (01.0) | −0.6 (0.9) | .17 |
| Change at 26 weeks, mean (SD) | −0.6 (1.2) | −0.8 (1.8) | −1.0 (1.6) | .44 | −0.4 (1.1) | −1.0 (0.8) | −0.9 (0.6) | .17 |
|  | Duration of diabetes <10 years | | | | Duration of diabetes ≥10 years | | | |
|  | Group 1 (n=44) | Group 2 (n=47) | Group 3 (n=44) | *P* value | Group 1 (n=43) | Group 2 (n=44) | Group 3 (n=47) | *P* value |
|  |  |  |  |  |  |  |  |  |
| 12 weeks, mean (SD) | 8.0 (1.4) | 7.6 (1.1) | 7.5 (0.9) | .42 | 8.2 (1.1) | 8.2 (1.3) | 7.9 (1.1) | .21 |
| 26 weeks, mean (SD) | 7.9 (1.4) | 7.8 (1.7) | 7.5 (1.1) | .60 | 8.1 (1.2) | 8.1 (1.2) | 8.0 (1.4) | .60 |
| Change at 12 weeks, mean (SD) | −0.6 (1.0) | −1.3 (1.7) | −1.3 (1.8) | .07 | −0.3 (0.9) | −0.4 (0.9) | −0.8 (1.0) | .02 |
| Change at 26 weeks, mean (SD) | −0.7 (1.3) | −1.1 (2.1) | −1.3 (1.9) | .30 | −0.4 (1.0) | −0.5 (0.9) | −0.7 (0.8) | .34 |
|  | BMI <25 kg/m^2^ | | | | BMI ≥25 kg/m^2^ | | | |
|  | Group 1 (n=24) | Group 2 (n=35) | Group 3 (n=29) | *P* value | Group 1 (n=63) | Group 2 (n=56) | Group 3 (n=62) | *P* value |
|  |  |  |  |  |  |  |  |  |
| 12 weeks, mean (SD) | 7.8 (1.3) | 7.9 (1.2) | 7.7 (1.2) | .59 | 8.2 (1.2) | 7.8 (1.2) | 7.7 (0.9) | .047 |
| 26 weeks, mean (SD) | 7.7 (1.3) | 7.9 (1.3) | 7.4 (1.2) | .06 | 8.1 (1.3) | 8.0 (1.6) | 8.0 (1.2) | .53 |
| Change at 12 weeks, mean (SD) | −0.6 (1.1) | −0.5 (1.3) | −0.7 (0.8) | .43 | −0.5 (0.9) | −1.1 (1.5) | −1.2 (1.7) | .004 |
| Change at 26 weeks, mean (SD) | −0.7 (1.2) | −0.5 (1.4) | −1.0 (0.8) | .05 | −0.5 (1.1) | −0.9 (1.8) | −1.0 (1.7) | .20 |
|  | A_1C_ level <8.5% | | | | A_1C_ level ≥8.5% | | | |
|  | Group 1 (n=48) | Group 2 (n=49) | Group 3 (n=48) | *P* value | Group 1 (n=39) | Group 2 (n=42) | Group 3 (n=43) | *P* value |
|  |  |  |  |  |  |  |  |  |
| 12 weeks, mean (SD) | 7.6 (0.8) | 7.5 (1.0) | 7.4 (0.7) | .45 | 8.8 (1.4) | 8.3 (1.3) | 8.1 (1.1) | .048 |
| 26 weeks, mean (SD) | 7.6 (0.8) | 7.6 (1.3) | 7.3 (0.7) | .25 | 8.5 (1.6) | 8.3 (1.6) | 8.3 (1.5) | .74 |
| Change at 12 weeks, mean (SD) | −0.3 (0.7) | −0.4 (0.9) | −0.6 (0.7) | .17 | −0.8 (1.2) | −1.4 (1.7) | −1.6 (1.8) | .07 |
| Change at 26 weeks, mean (SD) | −0.3 (0.7) | −0.3 (1.2) | −0.6 (0.7) | .25 | 8.5 (1.6) | 8.3 (1.6) | 8.3 (1.5) | .74 |
|  | C-peptide level <0.6 ng/mL | | | | C-peptide level ≥0.6 ng/mL | | | |
|  | Group 1 (n=8) | Group 2 (n=11) | Group 3 (n=11) | *P* value | Group 1 (n=79) | Group 2 (n=80) | Group 3 (n=80) | *P* value |
|  |  |  |  |  |  |  |  |  |
| 12 weeks, mean (SD) | 8.5 (1.1) | 8.6 (1.6) | 7.6 (1.0) | .21 | 8.1 (1.3) | 7.8 (1.1) | 7.7 (1.0) | .20 |
| 26 weeks, mean (SD) | 8.2 (0.9) | 8.3 (1.7) | 7.9 (1.2) | .39 | 8.0 (1.3) | 7.9 (1.5) | 7.8 (1.3) | .51 |
| Change at 12 weeks, mean (SD) | −0.6 (1.4) | −0.7 (1.2) | −0.7 (1.1) | .96 | −0.5 (0.9) | −0.9 (1.5) | −1.1 (1.5) | .008 |
| Change at 26 weeks, mean (SD) | −0.8 (1.2) | −1.0 (1.2) | −0.5 (0.7) | .45 | −0.6 (1.1) | −0.7 (1.7) | −1.0 (1.5) | .12 |
|  | Insulin treatment | | | | Noninsulin treatment | | | |
|  | Group 1 (n=30) | Group 2 (n=33) | Group 3 (n=39) | *P* value | Group 1 (n=57) | Group 2 (n=58) | Group 3 (n=52) | *P* value |
|  |  |  |  |  |  |  |  |  |
| 12 weeks, mean (SD) | 8.2 (1.2) | 8.3 (1.3) | 8.1 (1.3) | .66 | 8.0 (1.2) | 7.6 (1.1) | 7.5 (0.7) | .07 |
| 26 weeks, mean (SD) | 8.4 (1.3) | 8.4 (1.7) | 8.2 (1.5) | .90 | 7.8 (1.3) | 7.7 (1.3) | 7.5 (0.9) | .28 |
| Change at 12 weeks, mean (SD) | −0.6 (0.9) | −1.0 (1.9) | −1.1 (1.7) | .58 | −0.4 (1.0) | −0.8 (1.0) | −1.0 (1.2) | .004 |
| Change at 26 weeks, mean (SD) | −0.6 (1.2) | −0.9 (2.1) | −0.9 (1.7) | .92 | −0.6 (1.1) | −0.7 (0.4) | −1.0 (1.3) | .11 |
